# Patient safety improvement in the gastroenterology department: An action research

**DOI:** 10.1371/journal.pone.0289511

**Published:** 2023-08-15

**Authors:** Amir Sadeghi, Abbas Masjedi Arani, Hosna Karami Khaman, Arezoo Qadimi, Raziyeh Ghafouri

**Affiliations:** 1 Gastroenterology and Liver Diseases Research Center, Research Institute for Gastroenterology and Liver Diseases, Shahid Beheshti University of Medical Sciences, Tehran, Iran; 2 Department of Clinical Psychology, Medical School, Center for the Study of Religion and Health, Shahid Beheshti University of Medical Sciences, Tehran, Iran; 3 Student Research Committee, Urology Research Center, School of Medicine, Tehran University of Medical Sciences, Tehran, Iran; 4 Student Research Committee, School of Nursing & Midwifery, Shahid Beheshti University of Medical Sciences, Tehran, Iran; 5 Department of Medical and Surgical Nursing, School of Nursing & Midwifery, Shahid Beheshti University of Medical Sciences, Tehran, Iran; University of Sharjah College of Health Sciences, UNITED ARAB EMIRATES

## Abstract

**Background:**

Patient safety is a global concern. Safe and effective care can shorten hospital stays and prevent or minimize unintentional harm to patients. Therefore, it is necessary to continuously monitor and improve patient safety in all medical environments. This study is aimed at improving patient safety in gastroenterology departments.

**Methods:**

The study was carried out as action research. The participants were patients, nurses and doctors of the gastroenterology department of Ayatollah Taleghani Hospital in Tehran in 2021–2022. Data were collected using questionnaires (medication adherence tool, patient education effectiveness evaluation checklist, and medication evidence-based checklist), individual interviews and focus groups. The quantitative data analysis was done using SPSS (v.20) and qualitative data analysis was done through content analysis method using MAXQDA analytic pro 2022 software.

**Results:**

The majority of errors were related to medication and the patient’s fault due to their lack of education and prevention strategy were active supervision, modification of clinical processes, improvement of patient education, and promotion of error reporting culture. The findings of the research showed that the presence of an active supervisor led to the identification and prevention of more errors (P<0.01). Regarding the improvement of clinical processes, elimination of reworks can increase satisfaction in nurses (P<0.01). In terms of patient education, the difference was not statistically significant (P>0.01); however, the mean medication adherence score was significantly different (P<0.01).

**Conclusion:**

The improvement strategies of patient safety in Gastroenterology department included the modification of ward monitoring processes, improving/modification clinical processes, improvement of patient education, and development of error reporting culture. Identifying inappropriate processes and adjusting them based on the opinion of the stakeholders, proper patient education regarding self-care, careful monitoring using appropriate checklists, and presence of a supervisor in the departments can be effective in reducing the incidence rate. A comprehensive error reporting program provides an opportunity for employees to report errors.

## Introduction

Patient safety is a global concern [1, 2], which is even recognized in the Hippocratic Oath under the title "Do no harm" [3]. The concept of patient safety in health care systems means prevention of errors and the resulting injuries in care recipients [4]. Medical error is defined as "the failure of a planned action or the use of a wrong plan to achieve a goal" [5]. Medical errors (MEs) are one of the main factors in the quality of hospital services and patient safety in health care systems, especially in the developing countries [6].

Medical errors are the third cause of death in the United States, with more than 250,000 deaths annually [7]. Approximately, 25% of patients entering a health care facility experience some form of injury and 1% of these patients either die or suffer serious disabilities due to these errors [8]. As one of the main safety problems, medical errors impose a significant financial burden on health systems. Given the preventable nature of most medical errors, health policymakers and health care professionals must consider the associated financial dimension [9].

The annual costs of harmful medical errors in the United States are estimated at nearly 17 billion dollars [5] Medical errors lead to increased morbidity, disability, prolonged hospitalization [10, 11], increased costs of treatment [12] and even death [12, 13]. It is estimated that about 50% of medical errors are preventable in hospitalized patients [14]. Therefore, it is necessary to pay more attention to patient safety and prevention of medical errors [12, 15]. All types of medical errors should be mentioned, for example, drug errors, misdiagnosis (late diagnosis and non-diagnosis), infections, and bedsores, wrong technique, laboratory errors, surgical sample errors, errors related to surgical pathology specimens [12, 16].

Medical errors include a) not doing the right or doing the wrong b) mistakes in the implementation, and c) errors with possible or actual harm. Medical errors have different effects on medical staff, patients and health systems [5, 17]. In the case of employees, it leads to distress and emotional complications, depression, anger and suicide; and in patients, it leads to multiple injuries and increases the length of stay and death of the patient in hospitals [5]. Several studies have been conducted to further investigate bed falls, hospital-acquired infections, and medication errors. These results are important indicators of the quality of care [15, 16, 18].

Tabatabaee et al. found that the mean prevalence of medication error per each medical case was 2.42. Giving non-prescription medicine (47.8%) was the highest and using the wrong form of the drug (3.9%) was the lowest medication error and there was no statistically significant relationship between medication error and the age, gender and marital status of nurses but it had relation with employment status and night shift [15]. Also Jabarkhil et al. noted that the safety culture of the patients at the hospital was inappropriate, particularly in the eight dimensions of the patient safety culture, immediate intervention was necessary in Afghanistan [16].

Patients hospitalized in emergency [19], Intensive care [10, 20], internal [21] and gastroenterology [22–24] departments are more exposed to adverse events and complications of diagnostic or surgical procedures, misdiagnosis, and drug complications than other patients [25].

More than one half of these incidents can be prevented [26]. Among medical errors, medication errors are the most common errors. Medication error (ME) is any harm to the patient caused by inappropriate use of medication. MEs occur during prescription, transcription, prescription, compliance, and drug monitoring [27]. According to a systematic study, medication errors in hospitalized patients in the Middle Eastern countries range from 11 to 90% [28]. In total, 7000 doses of medication are prescribed daily in the world, while errors occur in at least 20% of cases [29]. In other words, out of every five cases of drug administration, an error occurs in one case [30]. Garcia et al. reported 39% of medication errors in drug administration [13]. In addition, Trakulsunti and Antoni et al. stated that medication errors cause harm to 1.3 million people in the United States of America annually [31].

It should be noted that these reports are only the tip of the iceberg, because most cases of medication errors are not reported due to various reasons such as the absence of an error reporting system and the fear of being blamed [32].

It is estimated that the prevalence of medical errors in Iran is high [5]. The prevalence of medication errors by nurses in Iranian hospitals is 53%, which varies from 17% to 88%. This high prevalence and inefficient reporting of medication errors in Iran has caused serious concerns about patient safety [33]. Khammarnia et al. (2021) estimated the overall prevalence of medical errors at 50% based on the results of a random effect model [6]. Mahmoudi et al. reported a high prevalence of errors in medical centers [19]. So, The patient safety culture in the hospital is inappropriate and requires urgent intervention [34] and there is a need for more studies in this field to achieve a medical care system with the highest standards [35]. Therefore, the current research was carried out with the aim of improving safety in gastroenterology departments.

## Methods

The study was an action research study that was conducted with the aim of improving safety in the gastroenterology department. The research was conducted in May 2021 until August 2022. The participants were patients, nurses and doctors in the gastroenterology department of Ayatollah Taleghani Hospital in Tehran. Data were collected using questionnaires (safe measures tools, patient education effectiveness evaluation checklist, and treatment compliance) and individual interviews and focus groups.

### Study design and setting

A research action cycle was carried out including four stages of planning, implementation, observation, and reflection. In the first stage of action research (planning) purpose was identification the threats and risk of patient safety in gastrointestinal department, so relevant questionnaires were used to identify threats and risk factors for patient safety. The medication evidence-based checklist tool due to the high prevalence of medication errors, the patient education effectiveness evaluation form and the treatment adherence tool for evaluate communication and patient education were used.

Based on the initial evaluation, the areas that could be improved and safety promotion strategies were identified through individual interviews and focus groups. Individual interviews was conduct with 18 nurses and 5 doctors in the gastroenterology department. Two focus groups was designed with nurses in the gastroenterology department and a focus groups was developed doctors and nurses.

In the second stage of the cycle (implementation stage), priorities were determined based on the opinion of managers and participants, and then an implementation plan was designed. In this stages for determining priorities, a focus groups designed with 2 head nurses and 4 supervisors. In the focus group, they reviewed the proposed safety promotion strategies and then for determining the priority of implementation of strategies, they ranked the priority of safety promotion strategies 1–4.

**Fig 1:**
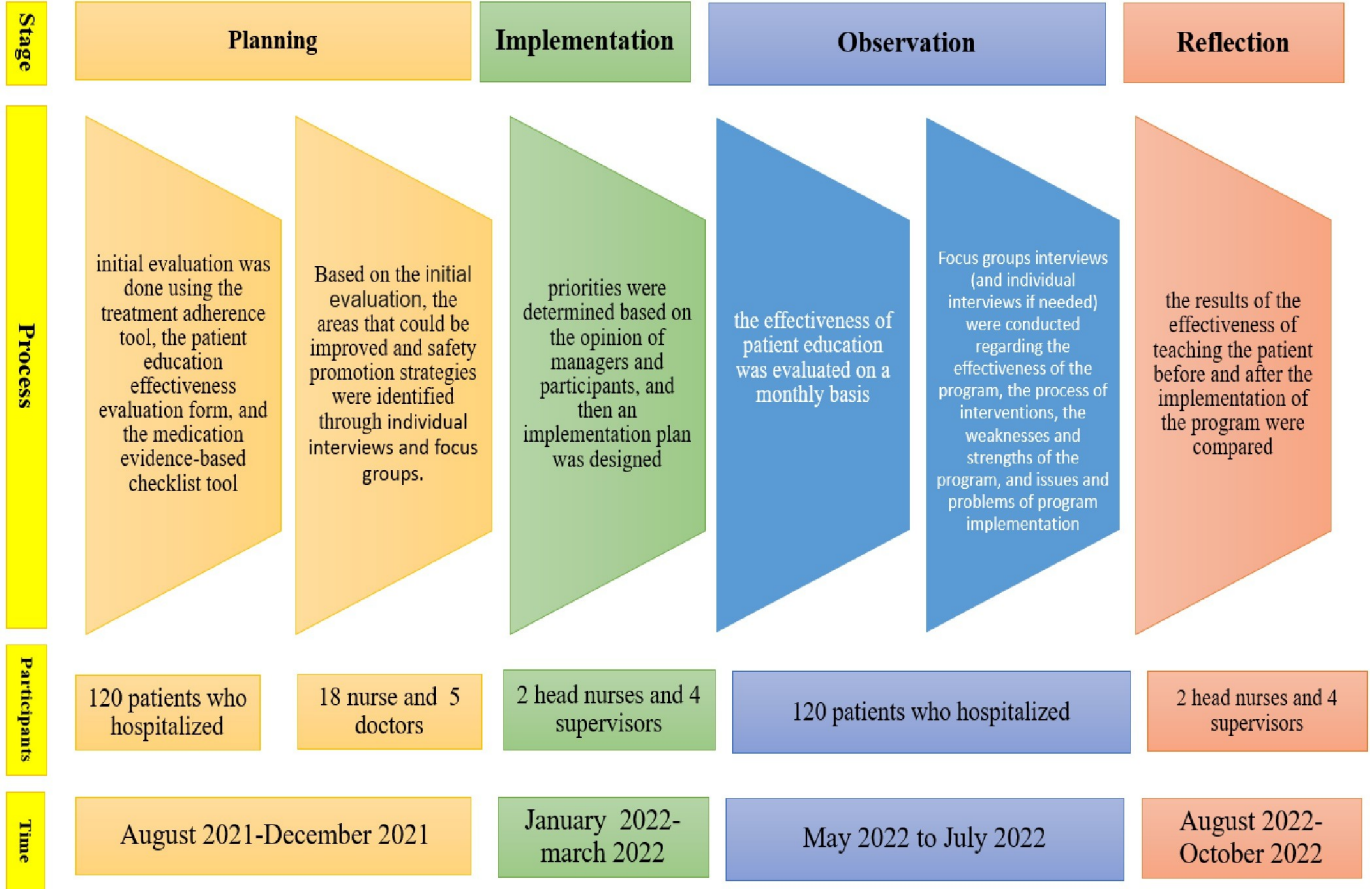
Study design.

Time-consuming processes such as rework in drug registration and reporting were eliminated, staff training programs were implemented, and the management team planned for more careful monitoring. In the third stage (observation), the effectiveness of patient education was evaluated. Focus groups interviews (and individual interviews if needed) were conducted regarding the effectiveness of the program, the process of interventions, the weaknesses and strengths of the program, and issues and problems of program implementation. In the fourth stage of the cycle (reflection), the results of the effectiveness of teaching the patient before and after the implementation of the program were compared. Fig 1 demonstrated the study design.

### Study participants

The participants were selected through a purposeful sampling method. The participants were patients, nurses and doctors in the gastroenterology department of Ayatollah Taleghani Hospital in Tehran. Patients were selected purposeful with inclusion criteria. The inclusion criteria of patients included hospitalization in the gastroenterology department for more than 24 hours in gastrointestinal ward or candidates for gastrointestinal procedures such as endoscopy, colonoscopy, EUS, ERCP from August 2021 to October 2021.

### Data analysis

To analyze the data collected using the questionnaire, descriptive and analytical statistics were used in SPSS (v.20) (IBM Corp., Armonk, NY) and to analyze the data from the interview, the qualitative content analysis method was used in MAXQDA 2022 (VERBI Software, Berlin, Germany).

Quantitative data was reported with descriptive statistics such as mean, standard deviation and percentage. For comparing the results of first stage with second stage, at the first evaluate the normality of data with Kolmogorov Smirnov Test (KS Test) in SPSS and then use The Mann–Whitney U Test because the data was not normal with KS Test (P>0.05).

### Data collection tool

Questionnaires (medication adherence tool, patient education effectiveness evaluation checklist, and medication evidence-based checklist), individual interviews and focus groups were used for data gathering.

#### Medication adherence tool

The medication adherence tool was designed by Kripalani et al. in 2009 to measure adherence to medications and prescription renewal in patients. The questionnaire consists of 12 items that examines two subscales namely medication adherence and prescription renewal (medication adherence subscale includes eight items and prescription renewal subscale includes four items). The items are four-alternative questions designed based on a 4-point Likert’s scale (always to never). The possible range for the instrument’s total score is 12 to 48 and the lower scores, the greater the adherence. Scores can be analyzed both on a continuous scale and dichotomously, i.e. 12 or >12. Validity and reliability of the original and Persian versions of this questionnaire have been confirmed [36]. The validity of the tool was checked and confirmed through face validity (qualitative) and content validity (quantitative and qualitative). The reliability of the tool was checked using Cronbach’s alpha (0.86), which indicated the appropriate reliability of the tool.

#### Patient education effectiveness evaluation checklist

Patient education effectiveness evaluation checklist was used for educating the participating patients (the form proposed by the Ministry of Health). The validity of the tool was checked and confirmed through face validity (qualitative) and content validity (quantitative and qualitative) and the reliability of the tool was checked using Cronbach’s alpha (0.79), which indicated the appropriate reliability of the tool.

#### Medication evidence-based checklist

In order to examine the medication process of nurses, the Medication evidence-based checklist [37] was used. This tool contains 10 items and is in the form of an observational checklist. The items are designed based on Likert’s four-point scale (always, often, sometimes, never) and the score range is from 10 to 40.

## Results

Patient who participate in this stage were 240 patients. They were 136 men and 104 women with mean (SD) age 57.08 (11.58) years old. Table 1 showed the Demographic Characteristics of the patients who participated in this study.

**Table 1:** Demographic characteristics of the participants.

Demographic Characteristics		Study Stage			
		First Evaluation		Second Evaluation	
		Mean	Standard Deviation	Mean	Standard Deviation
Age (Year)	Female	57.06	10.81	58.66	10.94
	Male	56.50	12.04	56.36	12.30
	total	56.73	11.50	57.43	11.69
Hospitalized Stay (Day)	All Participants	1.88	1.20	1.42	.86
		Count	percent %	Count	percent %
Gender	Female	50	41.7%	56	46.7%
	Male	70	58.3%	64	53.3%
	Total	120	100	120	100
Hospitalized Stay	Out patient	65	54.2%	87	72.5%
	At least 24 hours	25	20.8%	25	20.8%
	24–36 hours	16	13.3%	2	1.7%
	36–48 hours	7	5.8%	3	2.5%
	More than 48 hours	7	5.8%	3	2.5%
	Total	120	100	120	100
Couse of Hospitalized	GI Bleeding	10	8.3%	22	18.3%
	Endoscopy	20	16.7%	6	5.0%
	Colonoscopy	34	28.3%	36	30.0%
	Others	56	46.7%	56	46.7%
	Total	120	100	120	100

In the first stage of action research (planning), an initial assessment was done using the safety measures tool, and then the points that could be improved and ways to improve safety were identified through interviews. The interviews with nurses and doctors started with open questions and included the following:

- How do you think errors could be prevented?
- Do you have any suggestions to attenuate errors?
- If you are in charge of a ward or hospital, what plan will you adopt to identify errors?

Medication errors were checked using the Medication evidence-based checklist, and the common medication errors were the wrong prescription of drugs with the same shape, similar name, and mistakes in recording medication cards and calculating the correct dose of medication. As for patient education, the patient education effectiveness evaluation checklist was used and most of the cases were about self-care and taking medicine at the time of discharge and follow-up after discharge.

In the next stage, an individual interviews were conducted with 21 nurses with mean (SD) work experience 9.68 (5.82) years. After analyzing the data using the content analysis method, categories and suggested preventive strategies were identified. The evaluation time was also determined.

The prevention strategy included the modification of ward monitoring processes, improving/modification clinical processes, improvement of patient education, and development of error reporting culture (Table 2).

**Table 2:** Categories resulting from qualitative findings.

Subcategories	Categories	Suggested prevention subcategories	Priority	Evaluation time (month)	Evaluation
• Supervisor’s presence in the department • Continuous supervision • Having a supervisor in the department with defined criteria • Proper supervision	Active supervision	• Active monitoring of the supervisor’s presence in the department	2	1	• Evaluation of error • Supervisors reports • Evaluation of medication process
• Rework • Useless work and waste of time • Doing things in the traditional way • Lack of time and insurances	Improving /modification clinical processes	• Identification and elimination of reworks	1	1	• Evaluation of error • Employee Survey
• Using the facility and software for patient education • Outdated pamphlets • Face-to-face education	Improving the process of patient education,	• Using programs and software for patient education • Using facilities in patient education	3	3	• Patient education assessment • Medication adherence evaluation
• Sense of security for reporting • The possibility of reporting • Attitude to error reporting • An efficient error reporting system	Improving the culture of error reporting,	• Appropriate software for reporting errors • Improve the safety culture • Providing error reporting system	4	‐‐	‐‐

### Implementation of prevention strategy

#### Improving clinical processes

In the second stage of the cycle (implementation stage), since the most errors were in the registration of the drug card, the medication process was compiled using Cardex and removing the drug card was implemented under the strict supervision of the supervisor and clinical supervisors. Drugs with similar names and similar shapes were updated. Some of the clinical procedures are done in a traditional and repetitive way, such as writing prescriptions, cardex, medication cards, taking medicine and writing reports, which can be done faster and more accurately with the help of computer programs. It also saves time. On the other hand, having a good interaction between managers, nurses, doctors, pharmacists, and pharmacy managers can identify many mistakes before they happen and harm the patient.

Focus groups interviews (and individual interviews if needed) were conducted with the participants regarding the effectiveness of the program, the process of interventions, weaknesses and strengths of the program, and issues and problems of program implementation (observation stage). At the end, the results of the evaluation of the medication process were compared and feedback was given to the participants (reflection stage) (Table 3).

**Table 3:** The results of implementing the safety improvement program.

	Second evaluation	First evaluation	Evaluation	second Evaluation % / M(SD)	First Evaluation % / M(SD)	Result
**Active supervision**	July 2022	May, 2022	Evaluation of error	21/44%	16.84%	
	July 2022	May, 2022	Supervisors reports		‐‐	
	July 2022	March 2022	Evaluation of medication process	23.81 (3.36)	19.54 (3.93)	P_man_<0.01
**Clinical process improvement**	March 2022	May, 2022	Evaluation of error	21/44%	16.84%	
	March 2022	-	Employee Survey	82/90%	-	
**Patient education improvement**	March 2022	May, 2022	Patient education assessment	11.50 (3.58)	10.75 (4.44)	P_man_>0.01
	July 2022	May, 2022	Medication adherence evaluation	28.56 (4.57)	25.18 (5.22)	P_man_<0.01

#### Active supervision

As for active supervision in the planning stage, the proposed supervision solutions were collected using the managers’ opinions through interviews (planning stage). Selection criteria for supervisors were formulated and a training session was held for them. The supervisors monitored the departments for one month (implementation phase). Focus groups interviews (and individual interviews if needed) were conducted with the participants regarding the effectiveness of the program, the process of interventions, weaknesses and strengths of the program, and issues and problems of program implementation (observation stage). At the end, the results of the evaluation of the departments before and after the implementation of the program were compared and feedback was given to the participants (reflection stage) (Table 3).

#### Patient education

Common diseases in the department were identified and suitable training contents were prepared and after approval of the contents, pamphlets, clips and social media pages (Instagram) were prepared and made available to patients (implementation stage). The effectiveness of patient training was evaluated on a monthly basis, and they were interviewed about the way training was provided and their adherence to treatment was checked using relevant tools. In addition, focus groups interviews (and individual interviews if needed) were conducted with the participants (nurses) regarding the effectiveness of the program, the process of interventions, the weaknesses and strengths of the program, and the issues and problems of implementing the program (observation stage). In the end, the results of the effectiveness of the patient education before and after the implementation of the program were compared and feedback was given to the participants (reflection stage) (Table 3).

#### Designing an error reporting system

In total, 19 errors were reported in the gastrointestinal department in 2021 and according to the evaluation of the supervisors, it was found that many errors were not reported. One of the problems related to safety is the low error reporting rate. The proposed solution was to improve the culture of error reporting with the aim of learning from errors and designing an error reporting system.

## Discussion

This study was conducted with the aim of improving safety in a gastrointestinal department. The findings indicated that the modification of department monitoring processes, appropriate training, modification of clinical processes, improvement of patient education, and implementation of efficient error recording tools can be effective in improving safety. Regarding active supervision, the research findings showed that the presence of an active supervisor improved the effective identification and prevention of errors (P<0.01). Regarding the improvement of clinical processes, the elimination of rework led to an increase in satisfaction in nurses (P<0.01). In terms of patient education, despite the increase in the mean scores, the difference was not statistically significant (P>0.01); however, the mean medication adherence was significantly different (P<0.01).

### Improving clinical processes

As for medication administration, studies have emphasized that one of the challenges that requires special attention is the medication administration process [10, 38–41]. Medication error was the most frequent medical error. Estahbanati et al. stated that the insufficient diligence in nurses, using Cardex to record physicians’ orders, and the workload were the main factors in medication errors [5]. Tabatabaee et al. emphasized that training nurses, adopting an evidence-based care approach and creating interaction and coordination between nurses and pharmacists in the hospital can play an effective role in reducing the medication error of nurses [15]. Also Promoting patient safety culture can effectively reduce the medical errors [34]. Eliminating redundancies and traditional actions with effective interaction was one of the effective solutions that was associated with increasing the satisfaction of the participants.

### Active supervision

The findings showed that the presence of an active supervisor led to an effective identification and prevention of errors (P<0.01). Various factors, including managerial, environmental, and nursing care factors play a role in medical errors by nurses [5]. Despite the global efforts to convince people working in hospitals that reporting errors is the first step towards preventing medical errors, the reporting rate of medical errors is still very low [42]. Boostani et al. reported that 89% of patients experienced at least one ME during their stay in hospital [43]. One of the reasons is the absence of a clinical pharmacist in clinical visits. Considering that clinical pharmacists also supervise the medication administration process, they emphasized the presence of an active supervisor in the medication administration process and their results are consistent with the present study.

People tend to be reluctant to report their mistakes because of feedback from their peers and managers, including reprimands, legal actions, and the deterioration of their professional image in the workplace. In their study, Bairami & Taleghani used supervisors to identify errors and encourage employees to report errors. Based on the results, they stated that having a supervisor can be effective in promoting the culture of reporting medical errors [42]. Errors in human action are inevitable and act as an integral part of human reality [17, 19]. Even the most skilled experts may have MEs [3]. Reporting errors can help prevent similar incidents in the future [3, 17]. Therefore, given the effect of an active supervision on reporting and preventing errors, their results are in line with the results of the present study.

### Patient education

There were no significant improvements in patient education despite the increase in the mean scores (P>0.01). One of the reasons for this is the lack of time for training and the short evaluation period. It should be noted that in the field of self-care education, the lack of information in the process of treating patients causes anxiety and fear in the patient, and this factor can lead to many physical, mental, and psychological complications. Self-care training from the time of admission to the time of discharge of the patient can be of great help in the recovery process [44]. Therefore, the training should respond to the needs of the patients according to the disease and the treatment process.

### Designing an error reporting system

Despite tremendous efforts to encourage open communication, underreporting remains a major concern. A wide range of individual, organizational and cultural barriers prevent transparency in reporting. Developing a reporting culture requires introducing a persuasive approach that targets and removes such barriers [3]. Job satisfaction, learning from mistakes with a managerial approach, electronic prescribing, using systems based on preoperative checklists, and using root because analysis can lead to a reduction in medical errors. Various factors, such as the decentralized nature of the health care delivery system, lack of personnel, high workload, attitude of doctors and nurses towards patient safety, misinterpretation of prescriptions or inability to perform patient assessment, and lack of expertise in the staff are involved in the occurrence of medical errors [5].

Due to the fact that nurses have the largest contribution to care; therefore, they have a significant role in the occurrence or prevention of errors [45]. The most common problems in medical error reporting were the non-participation of doctors in the error reporting process, and structural (human and informational) problems [33]. Mahdavizadeh et al. reported that, among doctors and nurses, 57.1% and 49.4% had a little tendency to report errors respectively. Physicians tended to give only verbal warnings to their colleagues (36.8%). Nurses stated that they would report serious errors (32.4%). Fear of blame and punishment and fear of legal consequences were perceived as the most important barriers [3].

Poorolajal et al., stated that people’s lack of attention to the importance of medical errors and the lack of an effective reporting system for medical errors, and emphasis on the person who commits the error, were the reasons for reluctance to report medical errors [46]. Developing a systematic approach to the culture of error reporting and active participation in staff and identifying and knowing the causes that lead to medical errors can be helpful [19]. Evidence has shown that an anonymous, non-punitive critical incident reporting system can act as a powerful tool to identify the majority of medical errors and risk factors and may help prevent preventable adverse events. Such systems can turn threats into opportunities to learn from mistakes and identify strategies to prevent medical errors [46]. Also, Jabarkhil et al. emphasized the creation of a desirable organizational climate and the creation of a culture of error reporting for improving patient safety is necessary [16].

Nan et al. suggested using a platform of dynamic checklist applications, with which specialists can use the checklist to check the general condition of patients and prescribed drugs [14]. In their study, Mohanty et al., used MEDMARX to collect medication error event data [47]. Karami & Hafizi emphasized that developing strategies for designing an electronic monitoring system was effective in promoting error reporting; however, users may consider software programs as an extra workload [4]. Therefore, the practicality and simplicity of the programs should be considered in the design.

It is emphasized that due to the high incidence of errors, having corrective processes is one of the management priorities in every medical center. In addition, having a comprehensive error registration and reporting program provides an opportunity for safe reporting by employees and it also makes it possible to comprehensively examine medical errors and use them in learning from mistakes. Therefore, more research is needed on the advantages and limitations of using error recording systems.

## Conclusion

The most of medical errors in Gastroenterology department were related to medication and lack of the patient education and the improvement strategy of patient safety in Gastroenterology department included the modification of ward monitoring processes, improving/modification clinical processes, improvement of patient education, and development of error reporting culture. Identifying inappropriate processes and adjusting them based on the opinion of the stakeholders, proper patient education regarding self-care, careful monitoring using appropriate checklists, and presence of a supervisor in the departments can be effective in reducing the incidence rate. This should be one of the management priorities in the health system. Having a comprehensive error reporting program provides an opportunity for employees to report safety errors.

## Limitations

One of the most important limitations of the research was the outbreak of the Covid-19 epidemic, which was done by observing the necessary precautions in the bedside of the patients, and was used to check opinions using virtual groups in messaging software.

## Supporting information

S1 File (SAV)
